# A pilot study of a pharmacist-led prescribing program for final-year medical students

**DOI:** 10.1186/s12909-019-1486-1

**Published:** 2019-02-12

**Authors:** David A. Newby, Barrie Stokes, Anthony J. Smith

**Affiliations:** 1 Discipline of Pharmacy and Experimental Pharmacology, School of Biomedical Science and Pharmacy, Faculty of Health, University of Newcastle, Callaghan, NSW 2308 Australia; 2 School of Medicine and Public Health, Faculty of Health, University of Newcastle, Newcastle, Australia

**Keywords:** Pharmacist, Interprofessional teaching., Prescribing., Medical students.

## Abstract

**Background:**

Junior doctors undertake a significant amount of prescribing; however, they are not well prepared for this, and report they would like more training in their undergraduate courses. To address this we tested a pharmacist-led prescribing program for final-year medical students.

**Methods:**

Sixteen final-year students took part in the program. The program involved students writing prescriptions and getting feedback from clinical pharmacists, undertaking prescribing and calculation tutorials, and spending time in the pharmacy department. Evaluation included a pre- and post-assessment of their confidence and skills in prescribing, and a feedback session discussing the strengths and weakness of the program, and their perceptions about the role of pharmacists. Changes in the pre- and post-assessment of confidence and skills were examined with permutation and Mann-Whitney U tests.

**Results:**

There was a significant improvement in students’ confidence in prescribing, and a small but consistent improvement in prescribing skills. Of note, no student prescribed inappropriately and potentially harmfully after the program. Participants were positive about the program, and indicated a better understanding about the pharmacists’ role and their ability to support them as junior doctors.

**Conclusions:**

This study has shown the potential effect of a pharmacist-led prescribing program on the skills and confidence in prescribing by medical students. It provided an interprofessional teaching opportunity, preparing students for a team-based approach to patient management.

**Electronic supplementary material:**

The online version of this article (10.1186/s12909-019-1486-1) contains supplementary material, which is available to authorized users.

## Background

Prescribing medicines is the single largest medical intervention that physicians will use in their practice. In Australia over 280 million prescriptions are dispensed annually in primary care under the Pharmaceutical Benefits Scheme [1], while in the United Kingdom there are over 1 billion items dispensed annually under the National Health Service [2]. Although medicines play an important role in healthcare, prescribing of medicines is associated with significant risks. A review of medication errors in Australian hospitals found there were an average of five prescribing errors per patient per admission, of which 20–25% were clinical errors such as the wrong dose or wrong route of administration [3]. A review of prescribing errors before and after the introduction of an electronic prescribing platform found 1.4 to 1.8 moderate/major prescribing errors that could result in increased length of stay, permanent harm or death, were made per 100 bed days in a public mental health hospital [4].

Junior doctors write a significant number of prescriptions. Studies have found interns write approximately 20% of all prescriptions, of which for almost 1 in 5 they were the sole decision-maker [5]. Interns are more likely to self-initiate prescribing when on night shifts and weekends, a time when they were least likely to have support with their prescribing [5].

Despite the obvious need for good prescribing skills, junior doctors report they are not well prepared to prescribe. Studies have found less than a quarter of junior doctors feel equipped to write a correct hospital prescription, with just over half indicating they could choose an appropriate drug for a common condition, but a quarter or less could select the right dose or the appropriate dose frequency of a medicine [6]. A study of interns in New South Wales, Australia, found none of them were able to complete a prescribing task correctly based on a clinical case scenario [7]. A number of other studies internationally have also highlighted concerns about the preparedness of medical students to prescribe as junior doctors [8–10].

Pharmacists are well placed to help medical students gain skills in prescribing given their knowledge of pharmacology and therapeutics, and their understanding of the requirements for writing a safe, legal and appropriate prescription. While there are examples of pharmacist-led training of junior doctors that have resulted in a significant reduction in prescribing errors [11, 12] there are few examples of pharmacist-only structured teaching of undergraduate medical students that have evaluated both the effectiveness and the impression that students have about such training [13, 14].

Given the lack of preparedness of junior doctors to prescribe we pilot-tested a structured, pharmacist-led prescribing program for final-year medical students in the Joint Medical Program of the University of Newcastle, Australia, adapted from one developed at the Hull York Medical School [15]. The aim was to improve prescribing confidence and skills, and to improve medical students’ understanding of the role pharmacists can play in the management of patients.

## Methods

### Sample population

All final year students undertaking their eight-week Medicine rotations during July/August and September/October 2011 at three tertiary hospitals in New South Wales, Australia (Calvary Mater Newcastle, John Hunter and Gosford Hospitals) were invited to take part in the pilot study. Students were provided with information on what the program and the evaluation would involve and gave written consent to take part.

### Prescribing program structure

The eight-week prescribing program had several components. First, students maintained prescriptions of patients that were under the care of their medical team each week, using a special medicine chart that was identical to the National Inpatient Medication Chart used in all hospitals in Australia [16], but printed on yellow paper to avoid accidentally being used to administer medicines. Once a week the students met with a pharmacist in the hospital who reviewed their prescribing and provided feedback. Second, students undertook tutorials on prescribing and drug calculations conducted by the clinical pharmacist. These involved a series of case scenarios, based on common conditions faced by junior doctors [5], where the students had to either select and prescribe a medication for a condition (prescribing tutorials) or calculate and prescribe a dose of a medication for a patient (calculation tutorials). During the tutorials the students received immediate feedback from the pharmacist. Finally, the students spent one afternoon during their rotation in the pharmacy dispensary getting exposure to the dispensing process.

### Evaluation

The prescribing program was evaluated in a number of ways. First, students completed a questionnaire rating their confidence in a variety of prescribing areas in week one and week eight using a confidence scale adapted from a scale developed to rate junior doctor confidence [17] (1 = not confident, 2 = satisfactory but lacking confidence, 3 = confident in most cases but wanting more experience, 4 = fully confident in most cases). Second, students completed a prescribing exercise using a clinical scenario in week one, and the same exercise was repeated in the final tutorial in week eight. The appropriateness of their prescribing was assessed using a previously validated scale (1 = inappropriate and potentially harmful, 2 = inappropriate but not harmful, 3 = appropriate with reservations, 4 = appropriate) [6]. A clinical pharmacologist and a clinical pharmacist, blinded to which rotation and hospital the student was in, and whether the chart was a pre- or post-assessment, judged the prescribing independently. Any differences were settled by consensus. Third, the students completed a short questionnaire on their opinions about the impact of the program on their awareness of good prescribing, and their preparedness to prescribe as a junior doctor. Finally, the students participated in feedback sessions. These were conducted by one member of the research team (DN) and questions were asked to explore the positive and negative aspects of the program, and what the students learnt about the role of pharmacists through the program. The questions asked are provided in Additional file 1. The discussions were recorded, transcribed, and the transcripts were analysed for common themes.

### Statistical analysis

As this was a pilot study no formal sample size was calculated. Most comparisons were descriptive. Differences in the pre- and post-program assessment of confidence in prescribing, and the evaluation of the students prescribing skills before and after the program, were examined using permutation tests. The permutation test is a non-parametric test that assesses whether two distributions are significantly different from each other without making any assumptions about the shape of the distributions [18]. It involves repeated random sampling of the scores of the two groups and the *p*-value is the proportion of the permutations where the differences were larger than the observed test statistic. This provides a one-sided test in which the null hypothesis is that the difference between the mean scores of the pre- and post-prescribing program assessments is zero, and the alternative hypothesis is that the difference is positive, corresponding to a general improvement in the distribution of scores due to the prescribing program. A random sample of 10,000 permutations was used to allow the calculation of a *p*-value with a precision of 0.0001. Differences in the distribution of scores was also tested with the Mann-Whitney U test; the same conclusions were reached from both the Mann-Whitney U and permutation tests and therefore only the results for the permutation test are reported.

## Results

All 23 students undertaking their rotations at the three sites volunteered and consented to participate. However, only 16 students (70%) undertook most or all of the required activities and completed the pre- and post-course questionnaires and assessments. Of the 16 students who participated nine were female (56%) and the mean age was 24 years, which is similar to the age and sex distribution of students in the medical program [19]. Nine of the students undertook the program in the July/August rotation (56%) and seven in the September/October rotation (44%).

### Changes in confidence in prescribing

The baseline levels of confidence in generic skills relating to prescribing was low and similar across both rotations, with 66 to 100% of students in each rotation rating themselves as ‘not confident’ or ‘satisfactory but lacking confidence’ (Additional file 2). Therefore, the results for both rotations were pooled for analysis. After completing the program the students showed significant improvements in their confidence across all areas of the generic prescribing skills assessed (Table 1). Of particular note was that after the program all students rated themselves as ‘confident in most cases but would like more experience’ or ‘fully confident in most cases’ for writing an inpatient prescription compared to none of the students rating themselves ‘fully confident’, and 25% rating themselves ‘confident in most cases’ at baseline. Also, none of the students rated themselves as ‘not confident’ in any of the generic prescribing areas after the program, except for one student who rated him/herself as ‘not confident’ in writing an outpatient prescription.

**Table 1:** Changes in confidence in generic skills in prescribing before and after the program

	Percentage rating as fully confident/confident (n)^1^	
	Pre	Post^2^
Selecting appropriate medications for a condition	13% (2)	88% (14)
Writing an inpatient prescription	25% (4)	100% (16)
Writing an outpatient prescription	6% (1)	88% (14)
Taking a medication history	75% (12)	100% (16)
Identifying potential drug interactions	6% (1)	69% (11)
Identifying potential adverse drug reactions	19% (3)	69% (11)
Monitoring the effectiveness of a medication	19% (3)	81% (13)
Planning discharge medications	13% (2)	94% (15)

1. Students rating as either ‘Confident in most cases but would like more experience’ or ‘Fully confident in most cases’

2. All results *p* < 0.05 based on permutation test across the four rating categories compared to baseline

The students significantly improved their confidence in writing prescriptions, particularly for medications that were the focus of tutorials in the prescribing program including anticoagulation, pain management, and managing nausea and vomiting (Table 2). Similar improvements were seen in the student’s confidence in selecting, and monitoring for effectiveness of medicines for common conditions.

**Table 2:** Changes in confidence in writing prescriptions for common conditions

	Percentage rating as fully confident/confident in most cases (n)^1^					
	Selecting medicines		Writing prescriptions		Monitoring for efficacy	
	Pre	Post^2^	Pre	Post^2^	Pre	Post^2^
Night time sedation	13% (2)	69% (11)	13% (2)	81% (13)	19% (3)	88% (14)
Anticoagulation^3^	19% (3)	100% (16)	13% (2)	100% (16)	38% (6)	94% (15)
Managing diabetes	13% (2)	81% (13)	6% (1)	75% (12)	38% (6)	100% (16)
Pain management^3^	19% (3)	94% (15)	19% (3)	88% (14)	38% (6)	94% (15)
Managing constipation	31% (5)	75% (12)	13% (2)	94% (15)	50% (8)	100% (16)
Managing hypertension	13% (2)	75% (12)	6% (1)	88% (14)	50% (8)	94% (15)
Managing asthma	38% (6)	81% (13)	13% (2)	81% (13)	38% (6)	88% (14)
Managing nausea and vomiting^3^	25% (4)	88% (14)	13% (2)	81% (13)	44% (7)	88% (14)

1. Students rating as either ‘Confident in most cases but would like more experience’ or ‘Fully confident in most cases’

2. All results p < 0.05 based on permutation test across the four rating categories compared to baseline

3. Topics that were the focus of the prescribing program

### Prescribing skills using a clinical scenario

**Fig. 1:**
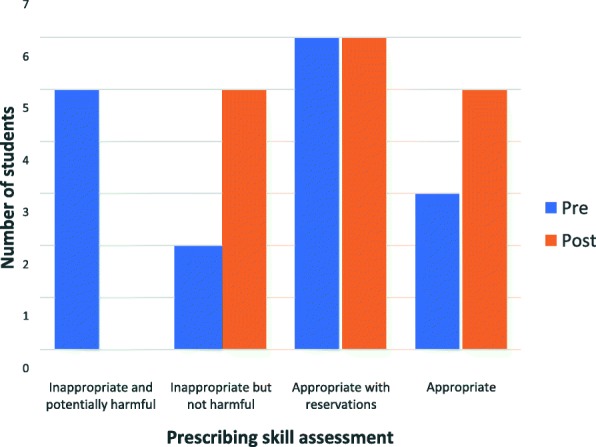
Rating of appropriateness of prescribing at baseline and at the end of the prescribing program

There were no differences in the baseline scores for prescribing appropriateness between those participants in the July/August rotation (Median 3; IQR [1, 3]) and those in the September/October rotation (Median 3; IQR [2, 3]). There was a small non-significant improvement in the appropriateness of the students’ prescribing from baseline to week eight. At the beginning the prescribing of nine students (56%) was rated as ‘appropriate with reservations’ or ‘appropriate’, which increased to 11 students (69%) after completing the program (*p* = 0.087 based on permutation test across the four rating categories). It is important to note that while the prescribing of five students (31%) was deemed ‘inappropriate and potentially harmful’ at baseline, none of the students’ prescribing was rated as ‘inappropriate and potentially harmful’ after finishing the prescribing module (Fig. 1).

### Perceptions of the prescribing program

All students at the end of the program agreed or strongly agreed with the statements that the program had improved their awareness of good prescribing practice in the writing of prescriptions, made them confident about the practical aspects of prescription writing, improved their awareness of the need to review the whole prescription in terms of the therapeutic appropriateness, and alerted them to the necessity of being able to undertake calculations of drug doses that they will prescribe for a patient. They also all agreed or strongly agreed that the prescribing program had helped prepare them to be able to undertake the basic prescribing required of doctors in their intern year. However, only 8 (50%) agreed or strongly agreed that their underpinning knowledge from years 1–4 was adequate to support the Year 5 program demands, with 2 (13%) disagreeing or strongly disagreeing with this statement.

During the feedback sessions at the end of the program, students indicated they were better prepared and more confident with prescribing as an intern. The students also valued the repeat practice with prescribing and the immediate feedback that they received. They indicated the program had enabled them to learn more about the role that pharmacists play in medication management, specifically the support they can provide to them as prescribers. The main negative comments were about the increase in workload that the program caused and the difficulty in fitting it in and around all the other demands of a very busy rotation.

## Discussion

This pilot study has shown the potential value of a pharmacist-led prescribing program in improving final-year student’s confidence and skills in prescribing. Despite the small number of students who participated, we found significant improvements in confidence in selecting medicines for common conditions, writing prescriptions, and monitoring the effectiveness of medicines. We also found improvements in their skills in prescribing, with no students prescribing inappropriately and unsafely after the program.

Using pharmacists who were working in the hospitals that the students were placed in added significant authenticity to the program and achieved our aim of interprofessional learning. Anecdotal feedback from pharmacists the following year was that students who undertook the program were presenting to pharmacy at the beginning of their intern year and requesting to speak with the pharmacists that had run the program for advice. Interprofessional learning opportunities within undergraduate healthcare degrees pose a number of difficulties, in particular timetabling opportunities for students to interact and learn. Opportunities for interprofessional teaching may be realised with delivery of the program by healthcare professionals from other areas of the health workforce. The potential role of pharmacists in undergraduate medical training was highlighted in a report prepared for the General Medical Council in the UK on prescribing errors of trainee doctors [20]. The report recommended that during practical placements, undergraduate medical students should spend time with pharmacists to better understand the prescribing process. Our prescribing program was grounded in providing authentic learning opportunities. An authentic learning environment situates tasks in the context of future use [21]. This allows students to experience the same problem-based challenges they will face in their future practice. As such our prescribing program complements the problem-based approach to teaching that is used in our medical program and by many medical schools across Australia and the world [22, 23].

A significant limitation of this study was the lack of a control group. This makes it more difficult to attribute any improvement in prescribing skills and confidence to the program, or to other experiences or teaching that they received during the Medicine rotation. However, enrolling a control group would have been difficult because the placements for students, other than the three large teaching hospitals that we undertook the program in, are largely in small, rural or regional hospitals, where students may get quite different experiences. Despite the lack of control there are a number of elements that suggest that the program was responsible for the improved confidence and skills in prescribing. First, the students who undertook the pilot program were in their last two rotations of their final year and had already undertaken a number of clinical attachments prior to the program. Despite this, both groups had low confidence and skills in prescribing at baseline. Even the last rotation group (September/October) showed similar low levels of skills and confidence in prescribing as the other rotation (July/August), despite an additional 8 weeks of clinical practice. This suggests that clinical rotations, without structured teaching in prescribing, do not significantly improve confidence or skills in prescribing. Second, prescribing teaching is not embedded in the Medicine rotation that the students were undertaking, with a large focus on achieving clinical skills (e.g. cannulation) and is largely assessed by a long-case presentation. Third, the change in confidence was so large it is difficult to attribute to incidental teaching on prescribing. Thus, we believe that the improvements seen are a result of the program.

The feedback from the students was overwhelmingly positive and supportive of the program. Our findings supports those of others that have found a positive impact of pharmacist-led teaching of junior doctors and medical students [12, 24]. Despite the potential for clinical pharmacist involvement in undergraduate medical education being discussed for nearly four decades [25], their role still appears limited. One reason for this could be a lack of formal recognition that teaching of other healthcare professionals, particularly students, is part of the role of a clinical pharmacist. The Society of Hospital Pharmacists of Australia *Standards of Practice for Clinical Pharmacy Services,* while recognising that pharmacists may provide education to other health professionals and students, focuses largely on the role clinical pharmacists play in the training of other pharmacists, pharmacy staff and pharmacy students [26]. Although the *Standards* recommend the establishment of clinical education pharmacist positions in hospitals, few if any of these exist in Australia. A study in the UK also found a lack of dedicated teaching roles as a limitation to greater involvement by clinical pharmacists in training medical students in prescribing [27]. The same authors found a lack of funding and resources for teaching, and no formal training on how to teach as important barriers to pharmacists’ delivering teaching to medical students. While a larger study is required to better quantify the impact that our program can have on improving prescribing, further research is also required to identify the institutional and personal barriers and enablers for clinical pharmacists that may exist that would allow such a program to be embedded in teaching at all hospitals.

## Conclusion

This pilot study has shown the potential effect that a pharmacist-led prescribing program can have on the confidence and skills in prescribing of final-year medical students. It also demonstrated that the program provides an excellent opportunity for interprofessional teaching that will better prepare the students for a team-based approach to managing patients. This model has the potential for wider application to teaching of other prescribers such as nurse practitioners.

## Additional files

Additional file 1: Feedback session questions. Questions asked during the feedback sessions at the end of the prescribing program. (PDF 83 kb)

Additional file 2: Baseline confidence ratings for generic prescribing skills for Rotation 5 and Rotation 6 students. Comparisons of the baseline ratings of confidence in generic prescribing skills between students in Rotation 5 and Rotation 6 (PDF 132 kb)

## Abbreviations

IQR: Interquartile range
UK: United Kingdom
