# Supplementary material for: A pilot study of a pharmacist-led prescribing program for final-year medical students
Source: BMC Med Educ. 2019 Feb 12;19:54. doi: 10.1186/s12909-019-1486-1 (PMC6373005; doi:10.1186/s12909-019-1486-1)
Supplement: Supplementary file 1 — Feedback session questions. Questions asked during the feedback sessions at the end of the prescribing program. (PDF 83 kb) [file 12909_2019_1486_MOESM1_ESM.pdf]

Focus group questions

What were the positive aspects of the prescribing program?
What have you learnt from the program about prescribing?
What have you learnt about the role of pharmacists in medication management?
What was an important take-away message from the program?
How useful was the face-to-face feedback?
How useful were the prescribing and drug dose calculation tutorials?
How useful was the formative OSCE session?

What were the possible negative aspects of the program?
How well prepared did you think you were to do the tasks?
How well did the previous 4 years prepare you for this task?
How was the timing?
How long did it take you to do all the tasks?
What parts did you find difficult to do?
Were any parts of it stressful?
Did you encounter any problems during the program? If so what?

How could the program be improved?
What aspects could be done better and how?
What other resources do you think you need to do this?
