# Supplementary material for: A pilot study of a pharmacist-led prescribing program for final-year medical students
Source: BMC Med Educ. 2019 Feb 12;19:54. doi: 10.1186/s12909-019-1486-1 (PMC6373005; doi:10.1186/s12909-019-1486-1)
Supplement: Supplementary file 2 — Baseline confidence ratings for generic prescribing skills for Rotation 5 and Rotation 6 students. Comparisons of the baseline ratings of confidence in generic prescribing skills between students in Rotation 5 and Rotation 6 (PDF 132 kb) [file 12909_2019_1486_MOESM2_ESM.pdf]

Baseline confidence ratings for generic prescribing skills for Rotation 5 and Rotation 6 students

	Percentage rating as 'not confident' or 'satisfactory but lacking confidence' (n)	
	Rotation 5 (N=9)	Rotation 6 (N=7)
Selecting appropriate medications for a condition	78% (7)	100% (7)
Writing an inpatient prescription	67% (6)	86% (6)
Writing an outpatient prescription	89% (8)	100% (7)
Taking a medication history	22% (2)	29% (2)
Identifying potential drug interactions	89% (8)	100% (7)
Identifying potential adverse drug reactions	78% (7)	86% (6)
Monitoring the effectiveness of a medication	89% (8)	71% (5)
Planning discharge medications	78% (7)	100% (7)
